# Hypertensive disorders of pregnancy and subsequent maternal cardiovascular health

**DOI:** 10.1007/s10654-018-0400-1

**Published:** 2018-05-19

**Authors:** Nienke E. Bergen, Sarah Schalekamp-Timmermans, Jolien Roos-Hesselink, Jeanine E. Roeters van Lennep, Vincent V. W. Jaddoe, Eric A. P. Steegers

**Affiliations:** 1 Department of Obstetrics and Gynaecology, Erasmus MC, Na 2918, P.O. Box 2040, 3000 CA Rotterdam, The Netherlands; 2 Department of Cardiology, Erasmus MC, Na 2918, P.O. Box 2040, 3000 CA Rotterdam, The Netherlands; 3 Department of General Medicine, Erasmus MC, Na 2918, P.O. Box 2040, 3000 CA Rotterdam, The Netherlands; 4 Department of Epidemiology, Erasmus MC, Na 2918, P.O. Box 2040, 3000 CA Rotterdam, The Netherlands; 5 Department of Paediatrics, Erasmus MC, Na 2918, P.O. Box 2040, 3000 CA Rotterdam, The Netherlands

**Keywords:** Blood pressure, Pregnancy, Hypertensive disorders, Cardiovascular follow-up

## Abstract

To examine associations between hypertensive pregnancy disorders and maternal cardiovascular disease (CVD) in later life. We examined the associations between blood pressure (BP) in pregnancy, gestational hypertension (GH) and preeclampsia (PE) with cardiovascular measurements 6 years after index pregnancy among 4912 women participating in the Generation R Study, the Netherlands. BP, left ventricular mass (LV mass), aortic root diameter (AOD), left atrial diameter, fractional shortening, and carotid-femoral pulse wave velocity (PWV). Early pregnancy systolic and diastolic BP were associated with more adverse maternal cardiovascular measurements and a higher incidence of chronic hypertension 6 years after pregnancy. GH was associated with a higher BP, a higher PWV, a larger AOD and an increased LV mass 6 years after index pregnancy. Compared to previous normotensive pregnancies these women had a sixfold increased risk to develop chronic hypertension after pregnancy (OR 6.6, 95% CI 4.6–9.5). Compared to women with a normotensive pregnancy, women with PE had a higher BP and a higher risk of chronic hypertension (OR 4.5, 95% CI 2.6–7.8) at follow-up. After adjustment for BMI at follow-up in all the analyses on GH, PE and cardiovascular measurements, effect estimates attenuated up to 65%, but remained significant. Both GH and PE are associated with markers of adverse maternal cardiovascular health after pregnancy with an increased risk of chronic hypertension. Women with GH and PE may be offered long-term cardiovascular follow-up incorporated in CVD risk management guidelines.

**Electronic supplementary material:**

The online version of this article (10.1007/s10654-018-0400-1) contains supplementary material, which is available to authorized users.

## Introduction

Differences between men and women exist regarding age-dependent onset, severity, symptoms and outcomes of cardiovascular disease (CVD) [1]. Increasing evidence has shown new cardiovascular risk factors exclusive to women related to pregnancy. These include gestational hypertension (GH) and preeclampsia (PE) [2]. The exact mechanisms by which these risk factors contribute to long term CVD risk have not been clarified. Women with GH or PE may exhibit the phenotype of metabolic syndrome or impaired endothelial function during, but also directly after, pregnancy which is also seen in later life [2, 3]. These include family history of diabetes mellitus, pregravid diabetes mellitus, a high total cholesterol/high-density lipoprotein cholesterol ratio (> 5), overweight and obesity, and elevated blood pressure status [3]. Exposure of women with this constitutional predisposition to the cardiovascular challenges of pregnancy may induce transient clinical disease that subsides after pregnancy (GH or PE) but is likely to re-emerge later in life as CVD [4, 5]. On the other hand it is also plausible that products of the dysfunctional placenta in hypertensive pregnancy disorders permanently compromise maternal cardiovasculature with long-lasting effects on cardiovascular health [6, 7]. In this respect pathophysiological studies may help to identify individuals after pregnancy with subclinical CVD as they might compose a target population for possible interventions before clinical signs and symptoms are evident. The aim of this study was to assess associations of maternal gestational blood pressure (BP) and hypertensive pregnancy disorders with cardiovascular outcomes 6 years after delivery.

## Methods

This study is embedded in the Generation R Study, a population-based cohort study [8]. The study protocol conforms to the ethical guidelines of the 1964 Declaration of Helsinki and its later amendments. The Medical Ethical Committee of the Erasmus Medical Centre Rotterdam approved the study and written consent was obtained from all participants. 5439 women with live born infants provided consent for postnatal analysis. We excluded women with missing or incomplete information on hypertensive pregnancy disorders (n = 110) or with known chronic hypertension before initial enrolment during pregnancy (n = 90). Also, women with cardiac abnormalities (n = 21), twin pregnancies (n = 34) and women being pregnant during their follow-up visit at the research centre (n = 257) or with missing data on medication use at follow-up (n = 15) were excluded, leaving 4912 participants for the analyses (Supplementary Information S1).

BP was measured in early pregnancy (median 13.2 weeks gestation, 90% range [10.6–16.9]), mid-pregnancy (median 20.5 weeks gestation, 90% range [19.1–22.4]) and late pregnancy (median 30.2 weeks gestation, 90% range [29.1–31.9]) and 6 years after delivery (90% range 5.7–7.2 years) with a validated automated digital oscillometric sphygmomanometer (OMRON Healthcare Europe B.V., Hoofddorp, the Netherlands) [9]. BP was measured in a research setting by trained research assistants wearing normal clothing (no white coats). Subsequently, mean arterial pressure (MAP) was derived. The presence of doctor diagnosed GH or PE was retrieved from hospital charts and was determined on the basis of the former 2001 criteria described by the International Society for the Study of Hypertension in Pregnancy [10, 11]. Information on chronic hypertension before onset of pregnancy was obtained through a questionnaire during pregnancy which was cross-checked with information from the original medical records and the Dutch obstetric database. Chronic hypertension at follow-up 6 years after pregnancy was defined as women using anti-hypertensive medication and/or having, in two subsequent readings, but at one single visit, a systolic BP above 140 mmHg or a diastolic BP above 90 mmHg. The value of two BP readings over a 5 min interval were documented for each participant. If women were having a systolic BP > 140 mmHg or diastolic BP > 90 mmHg in the first reading and also had a systolic BP > 140 mmHg or diastolic BP > 90 mmHg in the second reading we included them in the hypertension group. We are aware that international guidelines recommend ambulatory BP monitoring to define or confirm clinical diagnosis of hypertension. However ambulatory BP monitoring was not available. Instead we also performed a sensitivity analyses in which we labelled women as chronic hypertensive, only if they used BP medication at follow-up.

Data on cardiovascular outcomes were collected 6 years after index pregnancy at our research centre (range 4.9–7.0 years). Two-dimensional M-mode echocardiographic measurements were performed using the ATL-Philips Model HDI 5000 (Seattle, WA, USA) or the Logiq E9 (GE Medical Systems, Wauwatosa, WI, USA) devices. Aortic root diameter (AOD[sinus of Valsalva]) and fractional shortening (FS) were measured. Left ventricular mass (LV mass) was computed according to Devereux et al. [12]. Arterial stiffness was assessed by carotid-femoral pulse wave velocity (PWV) using an automatic non-invasive, validated device (Complior^®^; Artech Medical, Pantin, France). The distance between the recording sites at the carotid (proximal) and femoral (distal) artery was measured.

During pregnancy maternal height (cm) and weight (kg) were measured and body mass index (BMI) (kg/m^2^) was calculated. Identical measurements were obtained 6 years after index pregnancy. Pre-pregnancy BMI was established at enrolment through a questionnaire. Pre-pregnancy weight was highly correlated with the measured early pregnancy weight.

Information on maternal age, educational level, ethnicity, gravidity, self-reported pre-pregnancy weight, folic acid supplementation, smoking, pre-pregnancy history of chronic hypertension was available from questionnaires administered during index pregnancy. Information about gestational age at birth, birth weight and placental weight was obtained from medical records. Six years after index pregnancy we obtained information on subsequent pregnancies in between the index pregnancy and follow-up, anti-hypertensive medication use and educational level through questionnaires. Regarding anti-hypertensive medication use at follow-up, information on ATC-codes was not available.

### Statistical analysis

First, we performed a non-response analysis (Supplementary Information S2). Second, missing values were imputed using the multiple imputations procedure with five imputations and these datasets were analysed together. Third, differences in maternal characteristics were compared between women with hypertensive pregnancy disorders and those with normotensive pregnancies using Student’s *t* test, Mann–Whitney U test and Chi square test. Fourth, we used regression models to explore the combined effects of maternal BP in early and late pregnancy on vascular and cardiac outcomes, and chronic hypertension. In these analyses we divided maternal BP in early and late pregnancy into equal tertiles. We also performed conditional regression analyses to identify the independent associations of early, mid- and late pregnancy maternal BP, taking into account for their correlations, with vascular and cardiac outcomes and chronic hypertension. We constructed new systolic and diastolic BP variables, which are statistically independent from each other, by using standardized residuals obtained from linear regression models of maternal systolic and diastolic BP regressed on prior corresponding BP measurements (see for more details Supplementary Information S3) [13]. Fifth, using linear regression models, associations between women with GH or PE and women with a normotensive pregnancy and vascular and cardiac outcomes were assessed. These included; (1) basic model, adjusted for maternal age and visit interval; (2) confounder model, which in addition to model (1) included ethnicity, educational level, smoking, gravidity at follow-up, child’s sex; (3) BMI model, which included BMI at follow-up in addition to model (2). In the BMI model we observed whether changes in the effect estimates occurred after additional adjustment for BMI at follow-up. The difference between the effect estimates from model (2) and the effect estimates after adjustment for BMI was expressed as percentage change. The percentage change was calculated by the formula: 100 × (effect estimate_BMI_ − effect estimate_confounder_)/(effect estimate_confounder_ − 1). A 95% confidence interval for the percentage change of the effect estimate was calculated using a bootstrap method with 1000 resamplings [14, 15]. Using a similar approach multiple logistic regression models were used to examine the associations between hypertensive pregnancy disorders and normotensive pregnancies, and chronic hypertension at follow-up. Lastly, we carried out a sensitivity analysis by repeating the logistic regression analysis and defining chronic hypertension only on the basis of anti-hypertensive medication use at follow-up. Statistical analyses were performed using the Statistical Package for the Social Sciences version 21.0 for Windows (SPSS Inc, Chicago, IL, USA) and with R version 3.0.0 (libraries rmeta and metafor; The R foundation for Statistical Computing).

## Results

Table 1 shows maternal characteristics during index pregnancy and at follow-up. Women with a hypertensive pregnancy disorder had a higher BMI before and after the index pregnancy. They were also more often pregnant with their first child during the index pregnancy.

**Table 1:** Baseline characteristics by hypertensive pregnancy disorder (N = 4912)

	Normotensive pregnancies (n = 4612)	GH (n = 205)	PE (n = 95)
Maternal characteristics (pregnancy)			
Age at intake (years)	30.3 (5.1)	30.7 (4.9)	29.6 (5.3)
Height (cm)	167 (7.5)	168 (7.3)^a^	166 (7.5)^c^
Pre-pregnancy Body Mass Index (kg/m^2^)	22.7 (18.8–31.6)	25.2 (19.9–38.1)^a^	24.2 (19.2–38.9)^b^
Education, Higher (%)	42.5	44.9	32.6^b^
Ethnicity, European (%)	58.3	74.6^a^	55.8^c^
Gravidity at intake, Primigravida (%)	46.5	64.4^a^	69.5^b^
Smoking during pregnancy (%)	23.7	28.3	20.0^c^
Early pregnancy blood pressure (mmHg)			
Systolic (mmHg)	115 (12)	125 (13)^a^	121 (14)^b,c^
Diastolic (mmHg)	68 (9)	76 (11)^a^	74 (10)^b^
Mid-pregnancy blood pressure (mmHg)			
Systolic (mmHg)	116 (12)	127 (13)^a^	122 (14)^b,c^
Diastolic (mmHg)	67 (9)	76 (10)^a^	75 (90)^b^
Late pregnancy blood pressure (mmHg)			
Systolic (mmHg)	118 (12)	130 (13)^a^	128 (12)^b^
Diastolic (mmHg)	69 (9)	79 (9)^a^	79 (10)^b^
Birth characteristics			
Gestational age birth (weeks)	40.1 (37.1–42.1)	40.0 (37.1–42.0)	38.3 (31.2–41.1)^b,c^
Birth weight (g)	3438.6 (532.3)	3375.4 (593.6)	2823.2 (833.2)^b,c^
Male sex (%)	50.2	47.8	45.3
Maternal characteristics (follow-up)			
No subsequent pregnancies (%)	6.9	12.2^a^	16.8^b^
Body Mass Index (kg/m^2^)	24.6 (19.7–35.2)	27.6 (21.2–43.4)^a^	27.5 (20.0–43.8)^b^
Systolic blood pressure (mmHg)	119 (12)	130 (18)^a^	126 (15)^b^
Diastolic blood pressure (mmHg)	70 (10)	79 (13)^a^	78 (12)^b^
Mean arterial pressure (mmHg)	85 (73–104)	94 (78–125)^a^	92 (74–118)^b^
Pulse wave velocity (m/s)	7.6 (1.1)	7.8 (1.2)^a^	7.6 (1.1)
Fractional shortening (%)	36.9 (4.9)	37.5 (4.7)	37.3 (5.4)
Aortic root diameter (mm)	27.7 (2.8)	28.7 (2.9)^a^	27.9 (3.0)^c^
Left ventricular mass (g)	130.0 (30.9)	143.1 (34.6)^a^	133.0 (33.3)^c^
End diastolic left ventricular diameter (mm)	48.3 (4.0)	49.5 (4.1)^a^	48.7 (4.4)^c^
End diastolic left ventricular posterior wall thickness (mm)	8.0 (1.0)	8.4 (1.4)^a^	8.1 (1.4)
End diastolic interventricular septum thickness (mm)	8.1 (1.3)	8.4 (1.4)^a^	8.0 (1.4)
Anti-hypertensive medication (%)	1.2	6.3^a^	6.3^b^
Hypertension (%)^d^	4.9	23.4^a^	17.9^b^

Values represent means (SD), medians (90% range), or percentages. Measurements were performed in early pregnancy (median 13.2 weeks gestation, 90% range [10.6–16.9]), mid-pregnancy (median 20.5 weeks gestation, 90% range [19.1–22.4]) and late pregnancy (median 30.2 weeks gestation, 90% range [29.1–31.9]) and 6 years after delivery (90% range 5.7–7.2 years)

Differences in subject characteristics between groups were assessed using Student’s *t* test or Mann–Whitney U test for continuous variables and Chi square test for proportions

^a^Normotensive pregnancies versus GH with *P* value < 0.05

^b^Normotensive pregnancies versus PE with *P* value < 0.05

^c^GH versus PE with *P* value < 0.05

^d^Defined as women using anti-hypertensive medication and/or having, in two subsequent readings, a systolic or diastolic blood pressure above 140 or 90 mmHg, respectively

**Fig. 1:**
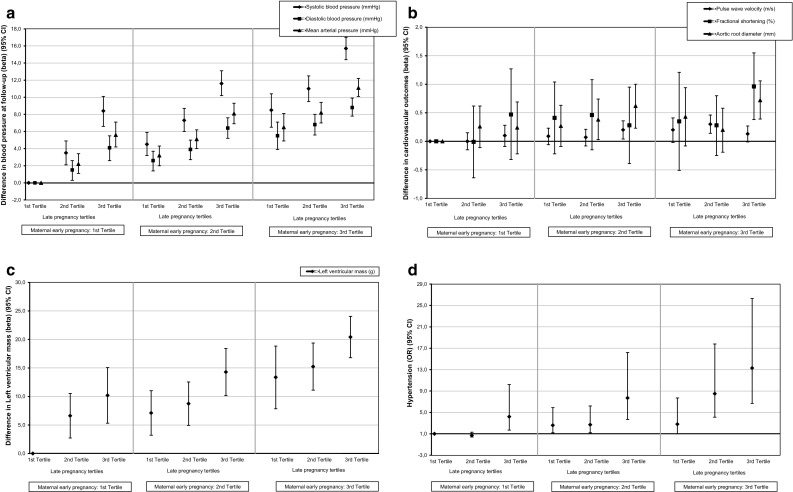
Combined associations of maternal early and late pregnancy blood pressure measures with cardiovascular outcomes (**a**–**c**) and the risk of hypertension (**d**) 6 years after pregnancy (n = 3551). Effect estimates or odds ratios (95% Confidence Interval) are from multivariable linear or logistic regression models, respectively. Results are from multiple imputed data. Women using anti-hypertensive medication at follow-up are excluded from regression analysis with cardiovascular outcomes (a,b,c) (n = 52). Hypertension (**d**) is defined as women using anti-hypertensive medication at follow-up and/or having, in two subsequent blood pressure readings, a systolic or diastolic blood pressure above 140 or 90 mmHg, respectively. Models are adjusted for maternal age at intake, visit interval, ethnicity, educational level, smoking, subsequent pregnancies between index and follow-up, and child’s sex

In Fig. 1 we presented the combined associations of maternal systolic and diastolic BP with cardiovascular outcomes and the risk of hypertension 6 years after pregnancy. As compared to women with a systolic or diastolic BP in the lowest tertiles during early and late pregnancy, those with a BP in the highest tertiles in early and late pregnancy had a higher systolic and diastolic BP, a higher MAP, a higher FS, a higher AOD, a higher LV mass and a higher risk of chronic hypertension 6 years after index pregnancy (all *P* values < 0.05 in confounder model).

Additionally, in the conditional analysis, which are presented in Supplementary Information S4 and S5, the independent associations of early, mid- and late pregnancy systolic and diastolic BP with cardiovascular outcomes and hypertension 6 years after pregnancy are shown. Early pregnancy systolic and diastolic BP were associated with BP, MAP, PWV, FS, AOD, LV mass and chronic hypertension 6 years after index pregnancy (all *P* values < 0.05 in confounder model).

The associations of early pregnancy BP appeared more strongly related to cardiovascular outcomes at follow-up than associations of mid- and late pregnancy BP.

The associations between hypertensive pregnancy disorders and vascular, cardiac outcomes and the risk of chronic hypertension at follow-up are presented in Tables 2 and 3.

**Table 2:** Associations of hypertensive pregnancy disorders with cardiovascular outcomes measured 6 years after pregnancy (n = 4837)

Outcome	Normotensive pregnancy n = 4556	GH		PE	
		n = 192 Difference (95% CI)	% Change (95% CI)	n = 89 Difference (95% CI)	% Change (95% CI)
Systolic blood pressure (mmHg)					
Basic model	Reference	10.9 (9.1, 12.7)**		6.4 (3.9, 8.9)**	
Confounder model	Reference	10.8 (9.1, 12.6)**		6.3 (3.9, 8.8)**	
BMI model	Reference	8.6 (6.8, 10.3)**	− 21.22 (− 24.57, − 18.29)**	4.4 (2.0, 6.8)**	− 28.90 (− 38.89, − 21.30)**
Diastolic blood pressure (mmHg)					
Basic model	Reference	8.4 (7.0, 9.8)**		6.9 (4.9, 8.9)**	
Confounder model	Reference	8.7 (7.3, 10.1)**		6.8 (4.8, 8.8)**	
BMI model	Reference	6.7 (5.4, 8.1)**	− 22.48 (− 25.40, − 19.44)**	5.1 (3.3, 7.0)**	− 23.08 (− 29.78, − 17.59)**
Mean arterial pressure (mmHg)					
Basic model	Reference	9.2 (7.8, 10.6)**		6.7 (4.7, 8.7)**	
Confounder model	Reference	9.4 (8.0, 10.8)**		6.6 (4.6, 8.6)**	
BMI model	Reference	7.3 (6.0, 8.7)**	− 21.99 (− 24.96, − 19.18)**	4.9 (3.0, 6.8)**	− 24.94 (− 31.88, − 18.97)**
Pulse wave velocity (m/s)					
Basic model	Reference	0.20 (0.01, 0.39)*		0.04 (− 0.22, 0.30)	
Confounder model	Reference	0.22 (0.03, 0.40)*		0.04 (− 0.23, 0.30)	
BMI model	Reference	0.20 (0.01, 0.38)*	− 7.98 (− 19.19, − 1.04)*	0.02 (− 0.24, 0.29)	NA
Fractional shortening (%)					
Basic model	Reference	0.36 (− 0.38, 1.09)		0.22 (− 0.85, 1.28)	
Confounder model	Reference	0.37 (− 0.37, 1.11)		0.19 (− 0.87, 1.26)	
BMI model	Reference	0.25 (− 0.49, 0.99)	NA	0.07 (− 1.00, 1.14)	NA
Aortic root diameter (mm)					
Basic model	Reference	1.01 (0.59, 1.43)**		0.24 (− 0.37, 0.85)	
Confounder model	Reference	0.90 (0.49, 1.32)**		0.26 (− 0.34, 0.87)	
BMI model	Reference	0.46 (0.05, 0.87)*	− 54.9 (− 70.75, − 44.29)**	− 0.10 (− 0.69, 0.50)	NA
Left ventricular mass (g)					
Basic model	Reference	13.29 (8.59, 17.98)**		3.46 (− 3.39, 10.31)	
Confounder model	Reference	12.03 (7.36, 16.71)**		3.93 (− 2.89, 10.75)	
BMI model	Reference	4.77 (0.45, 9.10)*	− 65.18 (− 78.08, − 55.65)**	− 3.15 (− 9.43, 3.13)	NA

Values are regression coefficients or % change (95% CI) and are based on linear regression models. % change represents the change in effect estimates after adjustment for BMI at follow-up with corresponding 95% CI. Estimates are from multiple imputed data

*Basic model* Adjusted for maternal age at intake and visit interval; *Confounder model* basic model and additionally adjusted for ethnicity, educational level, smoking, subsequent pregnancies between index and follow-up, and child’s sex; *BMI model* confounder model and additionally adjusted for BMI at follow-up

Women using anti-hypertensive medication at follow-up were excluded from these analyses (n = 75)

*P < 0.05; **P < 0.01

**Table 3:** Associations of hypertensive pregnancy disorders with the risk of hypertension 6 years after pregnancy (n = 4912)

Outcome			Normotensive pregnancy			GH				PE	
	n	%	n = 4612	n	%	n = 205 Odds ratio (95% CI)	% Change (95% CI)	n	%	n = 95 Odds ratio (95% CI)	% Change (95% CI)
Hypertension^**a**^	228	4.9		48	23.4			17	17.9		
Basic model			Reference			5.8 (4.1–8.3)**				4.4 (2.6–7.6)**	
Confounder model			Reference			6.6 (4.6–9.5)**				4.5 (2.6–7.8)**	
BMI model			Reference			4.7 (3.2–6.9)**	− 34.15 (− 38.57, − 29.83)**			3.5 (1.9–6.2)**	− 28.66 (− 37.18, − 20.48)**

Values are odds ratios or % change in odds ratio (95% CI) and are based on logistic regression models. % change represents the change in odds ratios after adjustment for BMI at follow-up with corresponding 95% CI. Estimates are from multiple imputed data

*Basic model* Adjusted for maternal age at intake and visit interval; *Confounder model* basic model and additionally adjusted for ethnicity, educational level, smoking, subsequent pregnancies between index and follow-up, and child’s sex; *BMI model* confounder model and additionally adjusted for BMI at follow-up

*P < 0.05; **P < 0.01

^a^Defined as women using anti-hypertensive medication at follow-up and/or having, in two subsequent readings, a systolic or diastolic blood pressure above 140 or 90 mmHg, respectively

Compared to women with a previous normotensive pregnancy, GH was strongly associated with both vascular and cardiac outcomes and the risk of chronic hypertension. Women with a history of GH had a 10.8 mmHg higher systolic BP (95% Confidence Interval (CI) 9.1–12.6), a 8.7 mmHg higher diastolic BP (95% CI 7.3–10.1), 0.22 m/s higher PWV (95% CI 0.03–0.40), a 0.90 mm larger AOD (95% CI 0.49–1.32) and a 12.03 g larger LV mass (95% CI 7.36–16.71) at follow-up. These women also had a sixfold higher risk to develop chronic hypertension six after index pregnancy (OR 6.6, 95% CI 4.6–9.5). Additional adjustment for BMI at follow-up showed a large attenuation of the effect estimates, especially in AOD and LV mass (total percentage change 54.9 and 65.18, respectively). However, these effect estimates remained significant. Sensitivity analyses, in which chronic hypertension was defined only on the basis of anti-hypertensive medication use at follow-up, showed similar results (Supplementary Information S6). Compared to women with a previous normotensive pregnancy, women with a history of PE had higher systolic and diastolic BP at follow-up (6.3 mmHg; 95% CI 3.9–8.8, and 6.8 mmHg; 95% CI 4.8–8.8, respectively). A history of PE was associated with a higher risk of chronic hypertension at follow-up (OR 4.5, 95% CI 2.6–7.8). Additional adjustment for BMI at follow-up showed that these associations attenuated by approximately 25%. Sensitivity analyses, in which chronic hypertension was defined only on the basis of anti-hypertensive medication use at follow-up, showed similar results (Supplementary Information S6). Cardiac outcomes were not associated with PE.

## Discussion

### Main findings

Women with a history of hypertensive pregnancy disorders exhibit a more unfavourable cardiovascular health after pregnancy compared to women with a previous normotensive pregnancy. Already 6 years after index pregnancy the prevalence of chronic hypertension shows an increased risk in both women with a history of GH or PE. The results further demonstrate that especially early pregnancy BP is strongly associated with the diagnosis chronic hypertension 6 years after pregnancy. Even though these results are strongly influenced by BMI at follow-up, effect estimates remained significant.

### Strengths and limitations

Strengths are the prospective data collection and large sample size. Complete information on pregnancies and pregnancy disorders that occurred in the years after the index pregnancy was not available. Instead we used gravidity at follow-up to account for pregnancies in between the index pregnancy and the follow-up measurement after 6 years. BP at study intake during pregnancy was higher in women with a history of GH than in women remaining normotensive throughout pregnancy. Pregnancy is associated with a physiologic decrease of BP which might suggest that non-random misclassification of the diagnosis chronic hypertension occurred in our study. Nevertheless, we think this is unlikely because information on chronic hypertension was cross-checked between multiple sources (maternal questionnaire in pregnancy and information from the original medical records and the Dutch obstetric database). Finally, we are aware of the fact that our approach to define chronic hypertension at follow-up might be suboptimal as 1) we defined chronic hypertension based upon BP measurements at one point in time at the 6 years follow-up visit (i.e. transient hypertension) and 2) international guidelines recommend that ambulatory BP monitoring should be used to define or confirm clinical diagnosis of hypertension because of prevalence of white coat hypertension. Transient hypertension is an established risk factor (i.e. early form) of chronic hypertension [16, 17]. Unfortunately, ambulatory BP monitoring was not available. Besides, BP was measured in a research setting by trained research assistants wearing normal clothing (i.e. no white coats). We also performed a sensitivity analyses in which we labelled women as chronic hypertensive, only if they used BP medication at follow-up. These analyses showed similar results (Supplementary Material S6).

### Interpretation

Studies show that hypertensive pregnancy disorders are associated with a woman’s risk of CVD [2]. This provides opportunities to identify women at risk early in their lives when it may be possible to alter their risk trajectory. It has been shown that measures of arterial stiffness and left ventricular function are increased during pregnancy among women with hypertensive pregnancy disorders [18, 19]. Arterial stiffness and left ventricular function are thought to be independent predictors of chronic hypertension and CVD. Also aortic root dilatation may be secondary to hypertension [18, 20, 21]. Franz et al. [22] showed differences in PWV after index pregnancy among former early, but not late, onset PE women. Likewise, Ghossein-Doha et al. [23] showed increased left ventricular mass indices and decreased cardiac diastolic function among a population of mainly severe (early onset) PE postpartum as proxy for hypertension. We observed increased PWV 6 years after index pregnancy among women with a history of GH. Similarly, women with a history of GH had an increased LV mass, a larger AOD, a higher BP and a higher risk of chronic hypertension. Interestingly, no differences were seen when comparing these cardiovascular measurements between women with PE and normotensive women, with the exception of an increased risk of chronic hypertension. Regrettably, GH has only rarely been included in postpartum research and despite evidence that differences in CVD risk exist between women with severe early-onset PE, mild late-onset PE and GH, most studies do not differentiate between the subgroups of hypertensive pregnancy disorders. One large longitudinal study which differentiated between (mild) PE and GH found that both GH and PE were associated with greater CVD risk factors. However after controlling for various confounders, results for PE were not significant anymore [24]. In a study by Wikström et al. [25] the risk of developing ischaemic heart disease was higher in women with severe PE compared with GH and mild PE [25, 26]. This was similar to results reported by Lykke et al. [27]. Women with mild PE had a four-fold higher risk of chronic hypertension. However, this risk increased up to sixfold in women with a history of GH and severe PE, respectively [25–27]. Similar patterns in mean BP, usage of anti-hypertensive medication and chronic hypertension were reported by Verbeek et al. [26] with the highest incidence of chronic hypertension in severe PE followed by GH and finally mild PE. In our study the majority of preeclamptic women had mild PE (92%) with only eight women suffering from severe PE. These results strengthen our findings on GH showing that not only severe PE women exhibit an increased risk of CVD but also that women with GH are at increased risk of an adverse cardiovascular health profile after pregnancy. However, the results could also indicate that, for women with GH, this phenotype already existed prior to the index pregnancy. Women with GH should in either case not be excluded from (secondary) preventive interventions. A meta-analysis found that lifestyle interventions may alter cardiovascular risk after a history of PE up to 13% with relatively simple intervention measures [28]. These may include exercise, dietary counseling and support for smoking cessation assistance.

Causal pathways relating hypertensive pregnancy disorders to chronic hypertension and CVD are unclear. One hypothesis focuses on common risk factors including among others obesity, chronic hypertension and genetic constitution [29]. Both PE and atherosclerosis arise from vascular inflammation with endothelial dysfunction. It has been also been hypothesized that hypertensive pregnancy disorders worsen pre-existing subclinical CVD risk factors already present before index pregnancy or even induce de novo risk [29]. A large population-based study showed that most CVD risk factors remain higher after PE following adjustment for pre-pregnancy values [30]. It is possible that products of the dysfunctional placenta in PE could permanently compromise maternal cardiovasculature [28]. A study demonstrated that increased sensitivity to infused Angiotensin II exists in the postpartum state in women with a history of new-onset hypertension in pregnancy and that this increased sensitivity to Angiotensin II is present in the vasculature and in the adrenal glands, with a suggestion of sFlt-1 responsiveness.[31] This study also showed that women with a history of new-onset hypertension in pregnancy were unable to modulate a response to infused Angiotensin II on the basis of salt intake. They suggested a dysregulation of the renin-angiotensin system.[31] Another study suggested persistence of left ventricular geometrical changes that herald the development of chronic hypertension [23]. In our study we observed significant associations regarding early pregnancy BP and all cardiac and vascular outcomes and the risk of chronic hypertension. Also the combined associations of maternal BP during early and late pregnancy were consistently associated with vascular, cardiac and hypertensive outcomes at follow-up. These findings may corroborate those of prior research supporting the theory that hypertensive pregnancy disorders share pathophysiology already programmed before the challenge of pregnancy that ultimately leads to CVD. However, they also cannot reject the hypothesis that hypertensive pregnancy disorders may cause permanent vascular damage thereby contributing to CVD risk.

## Conclusions

Hypertensive pregnancy disorders are associated with an adverse cardiovascular health profile and an increased risk of chronic hypertension 6 years after the index pregnancy. It is important to assess both GH and PE when assessing chronic hypertension and CVD risks. Women with GH and PE may be offered long-term cardiovascular follow-up incorporated in CVD risk management guidelines. BP profiles measured from early pregnancy onwards might help to further distinguish women at risk of future chronic hypertension and CVD.

## Electronic supplementary material

Below is the link to the electronic supplementary material.

Supplementary material 1 (DOCX 39 kb)

Supplementary material 2 (DOCX 20 kb)

Supplementary material 3 (DOCX 19 kb)

Supplementary material 4 (DOCX 141 kb)

Supplementary material 5 (DOCX 58 kb)

Supplementary material 6 (DOCX 31 kb)
