# Supplementary material for: Hypertensive disorders of pregnancy and subsequent maternal cardiovascular health
Source: Eur J Epidemiol. 2018 May 19;33(8):763–71. doi: 10.1007/s10654-018-0400-1 (PMC6061134; doi:10.1007/s10654-018-0400-1)
Supplement: Supplementary file 1 — Supplementary material 1 (DOCX 39 kb) [file 10654_2018_400_MOESM1_ESM.docx]

**Supplementary Information S1** Flowchart

**N = 8198**

Women enrolled during pregnancy

**N = 4912**

**Population for analysis**

Women with available information on hypertensive pregnancy disorders and postnatal follow-up data

No Anti-hypertensive drug use *n* = 4837

**n = 15**

Excluded women with missing data on antihypertensive drug use at follow-up

**n = 291**

Excluded twins (n=34) and women being pregnant during their visit at the research center (n=257)

**n = 21**

Excluded women having cardiac abnormalities

**n = 200**

Excluded women without information on hypertensive pregnancy disorders (n=110) and with chronic hypertension before their initial enrolment in the Generation R Study (n=90)

**n = 2759**

Excluded women without postnatal follow-up data

**n = 5439**

Women with postnatal follow-up
