# Supplementary material for: Hypertensive disorders of pregnancy and subsequent maternal cardiovascular health
Source: Eur J Epidemiol. 2018 May 19;33(8):763–71. doi: 10.1007/s10654-018-0400-1 (PMC6061134; doi:10.1007/s10654-018-0400-1)
Supplement: Supplementary file 2 — Supplementary material 2 (DOCX 20 kb) [file 10654_2018_400_MOESM2_ESM.docx]

**Supplementary Information S2** Non-response analysis for maternal follow-up data six years after pregnancy^*^

|  | **Follow-up at six years** | **Loss to follow-up at six years** |  |
| --- | --- | --- | --- |
|  | n = 5439 | n = 2759 | *P*-value^††^ |
| **Maternal characteristics** |  |  |  |
| Age at intake (years) | 30.2 (5.1) | 28.2 (5.5) | <0.001 |
| Gestational age at intake (weeks) | 13.9 (10.8-22.6) | 14.5 (10.8-23.8) | <0.001 |
| Height (cm) | 166.6 (7.5) | 166.1 (7.3) | 0.01 |
| Pre-pregnancy weight (kg) | 66.5 (13.3) | 65.6 (13.3) | 0.01 |
| Weight at intake (kg) | 69.6 (13.1) | 69.3 (14.0) | 0.43 |
| Pre-pregnancy Body Mass Index (kg/m^2^) | 22.9 (18.9-32.6) | 22.6 (18.2-33.1) | 0.09 |
| Body Mass Index at intake (kg/m^2^) | 24.1 (19.6-33.7) | 24.1 (19.1-34.4) | 0.59 |
| Systolic blood pressure at intake (mmHg) | 115.7 (12.3) | 114.7 (12.4) | 0.001 |
| Diastolic blood pressure at intake (mmHg) | 68.2 (9.5) | 67.5 (9.8) | 0.002 |
| Gravidity (%)^†^ |  |  |  |
| 1 | 263 (48.0) | 1165 (42.2) | <0.001 |
| ≥2 | 2792 (51.3) | 1543 (55.9) |  |
| Educational level (%)^†^ |  |  |  |
| None/Primary | 500 (9.8) | 386 (16.5) | <0.001 |
| Secondary | 2301 (45.3) | 1224 (52.4) |  |
| Higher | 2279 (44.9) | 728 (31.1) |  |
| Ethnicity (%)^†^ |  |  |  |
| Dutch/European | 3172 (59.7) | 1142 (47.3) | <0.001 |
| Non-European | 2144 (40.3) | 1271 (52.7) |  |
| Smoking (%)^†^ |  |  |  |
| No | 3563 (73.4) | 1612 (69.2) | <0.001 |
| Yes | 1291 (26.6) | 716 (30.8) |  |
| **Pregnancy complications** |  |  |  |
| Gestational hypertension (%)^†^ | 226 (4.4) | 79 (3.1) | 0.01 |
| Preeclampsia (%)^†^ | 106 (2.1) | 73 (2.9) | 0.04 |
| **Birth and infant characteristics** |  |  |  |
| Gestational age (weeks) | 40.1 (36.9-42.0) | 40.0 (36.1-42.1) | <0.001 |
| Birth weight (g) | 3410.4 (553.9) | 3346.1 (599.3) | <0.001 |
| Male sex (%)^†^ | 2714 (49.9) | 1424 (51.7) | 0.14 |

^*^*Values are means (standard deviation) or medians (90% range). ^†^Values are observed numbers and valid percentages. ^††^Differences in subject characteristics between the groups were evaluated using T-test or Mann Whitney-U test for continuous variables and chi-square tests for proportions.*
