# Supplementary material for: Hypertensive disorders of pregnancy and subsequent maternal cardiovascular health
Source: Eur J Epidemiol. 2018 May 19;33(8):763–71. doi: 10.1007/s10654-018-0400-1 (PMC6061134; doi:10.1007/s10654-018-0400-1)
Supplement: Supplementary file 3 — Supplementary material 3 (DOCX 19 kb) [file 10654_2018_400_MOESM3_ESM.docx]

**Supplementary Information S3**

**Conditional regression models**

Conditional regression models were used to examine the independent associations of maternal blood pressure (BP) development in each period of pregnancy with BP, cardiovascular outcomes and the risk of hypertension six years after index pregnancy, taking into account the correlation between maternal BP in early, mid- and late pregnancy.(1) For these analyses, we constructed new systolic and diastolic BP variables, which are statistically independent from each other, by using standardized residuals obtained from linear regression models of maternal systolic and diastolic BP regressed on all prior corresponding BP measurements: systolic BP is used as an example.(1) Systolic BP in early pregnancy is the starting point. Conditional change in systolic BP from early to mid-pregnancy is equivalent to the standardized residuals resulting from the linear regression model of mid-pregnancy BP on early pregnancy BP. Accordingly, the conditional change in mid-pregnancy BP to late pregnancy BP is given as the standardized residuals obtained from regressing late pregnancy BP on both mid-pregnancy BP and early pregnancy BP simultaneously.(2) By this approach, strong correlations are removed between systolic or diastolic BP measures in different periods in pregnancy which allows simultaneous inclusion of these related variables into one regression model. Subsequently, the influence of systolic or diastolic BP in specific periods in pregnancy can then be assessed in comparison with, and adjusted for, systolic or diastolic BP in the other pregnancy intervals.

*1.Keijzer-Veen MG, Euser AM, van Montfoort N, Dekker FW, Vandenbroucke JP, Van Houwelingen HC. A regression model with unexplained residuals was preferred in the analysis of the fetal origins of adult diseases hypothesis. J Clin Epidemiol. 2005;58:1320-4.*

*2.Harvey NC, Mahon PA, Kim M, et al. Intrauterine growth and postnatal skeletal development: findings from the Southampton Women's Survey. Paediatr Perinat Epidemiol. 2012;26:34-44.*
