# Supplementary material for: Hypertensive disorders of pregnancy and subsequent maternal cardiovascular health
Source: Eur J Epidemiol. 2018 May 19;33(8):763–71. doi: 10.1007/s10654-018-0400-1 (PMC6061134; doi:10.1007/s10654-018-0400-1)
Supplement: Supplementary file 4 — Supplementary material 4 (DOCX 141 kb) [file 10654_2018_400_MOESM4_ESM.docx]

**Supplementary Information S4** Associations of systolic (a,b) and diastolic (c,d) blood pressure measures in pregnancy with cardiovascular outcomes six years after pregnancy from conditional analyses (n = 3551)


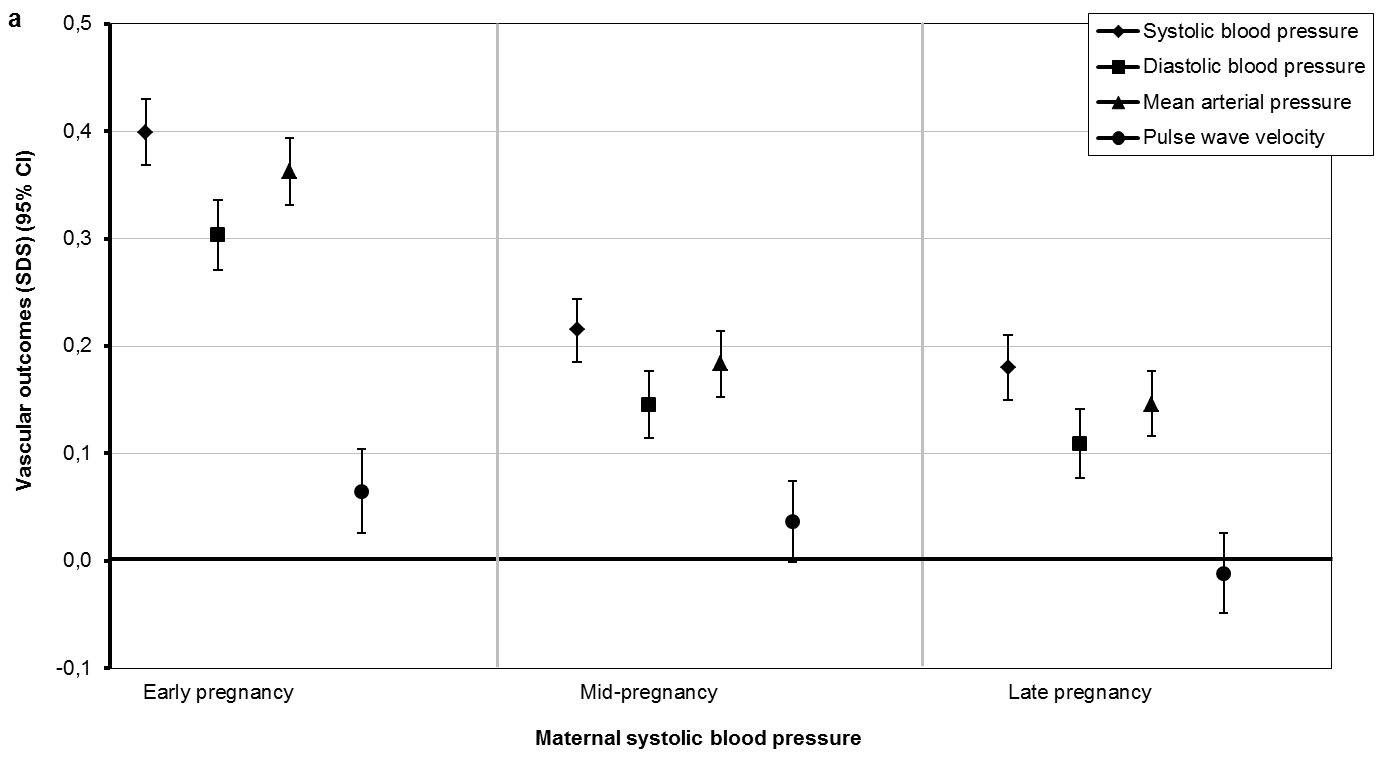

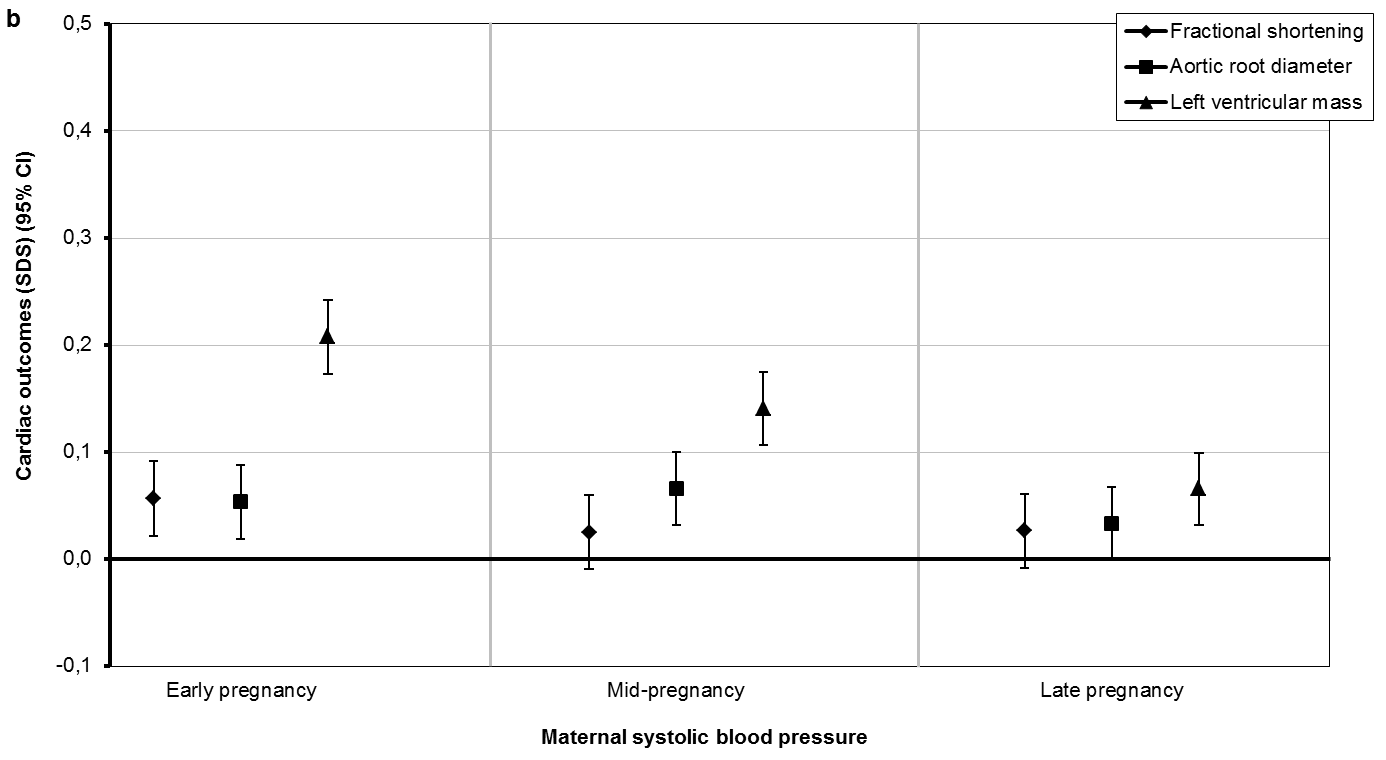


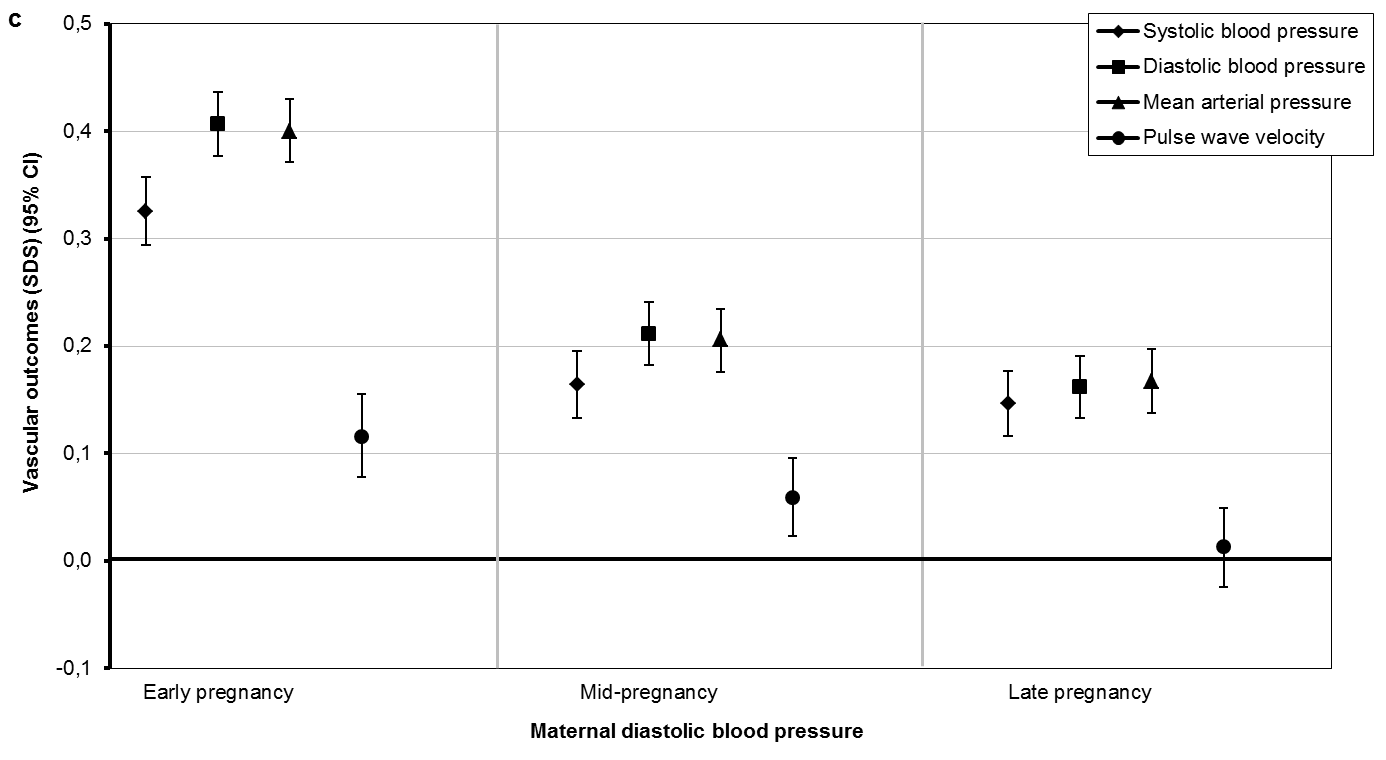

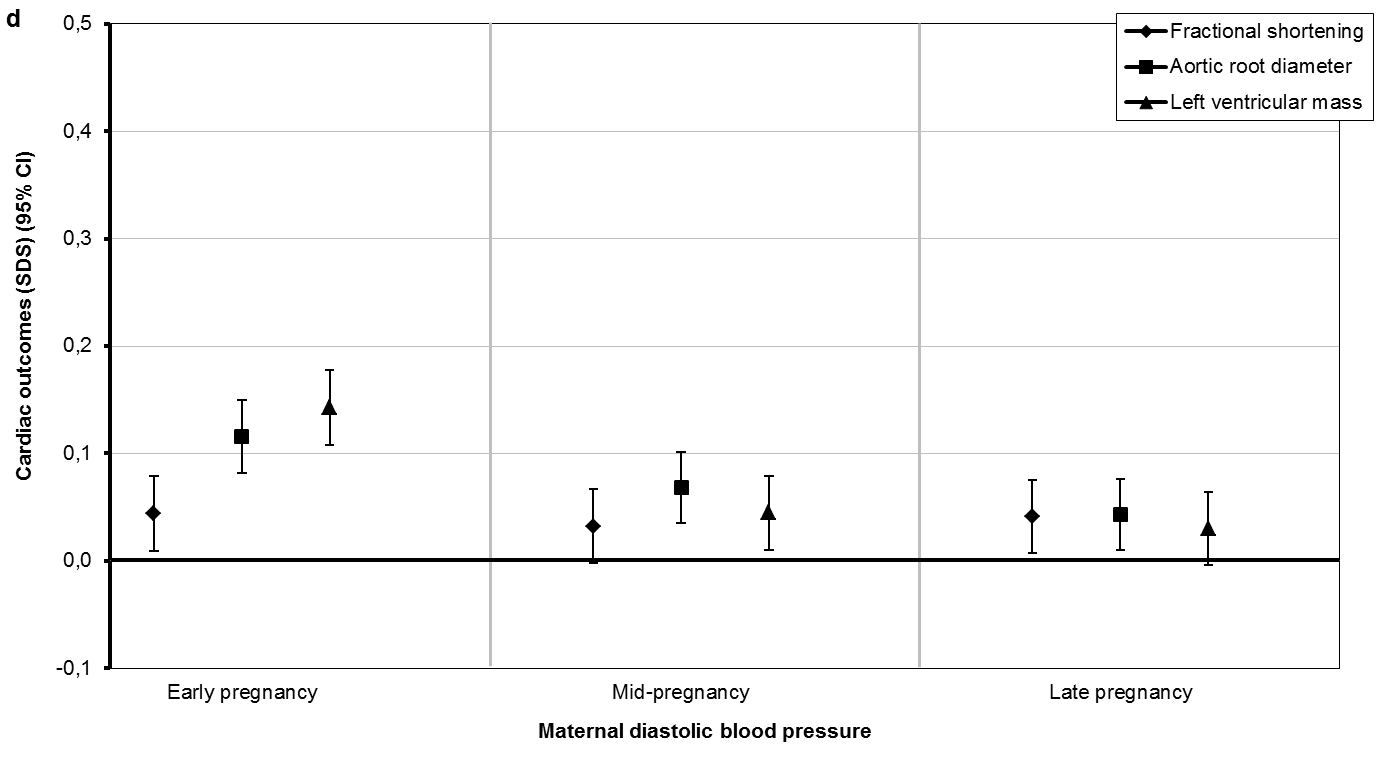


*Values are regression coefficients (95% Confidence Interval) from multivariable linear regression models and reflect the difference in cardiovascular outcomes per SDS change in early pregnancy systolic and diastolic blood pressure and per SDS change in standardised residual change in systolic and diastolic blood pressure in mid- and late pregnancy from conditional regression analyses (see for details of conditional regression models* ***Supplementary Information S3****). Women using anti-hypertensive medication are excluded from these analysis (n = 52). Models are adjusted for maternal age, visit interval, ethnicity, educational level, smoking, subsequent pregnancies between index and follow-up and child’s sex.*
