# Supplementary material for: Hypertensive disorders of pregnancy and subsequent maternal cardiovascular health
Source: Eur J Epidemiol. 2018 May 19;33(8):763–71. doi: 10.1007/s10654-018-0400-1 (PMC6061134; doi:10.1007/s10654-018-0400-1)
Supplement: Supplementary file 5 — Supplementary material 5 (DOCX 58 kb) [file 10654_2018_400_MOESM5_ESM.docx]

**Supplementary Information S5** Associations of systolic (a) and diastolic (b) blood pressure measures in pregnancy with hypertension six years after pregnancy from conditional analyses (n = 3551)


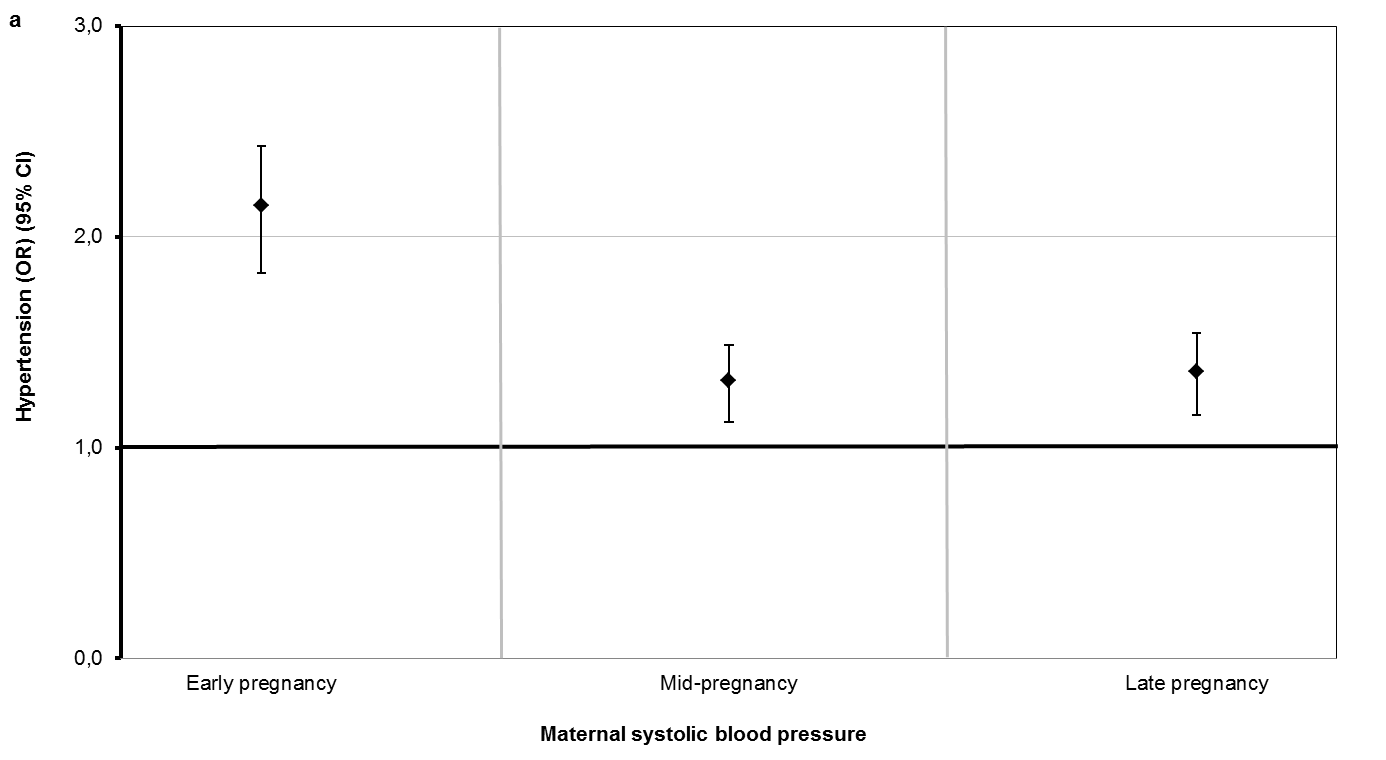


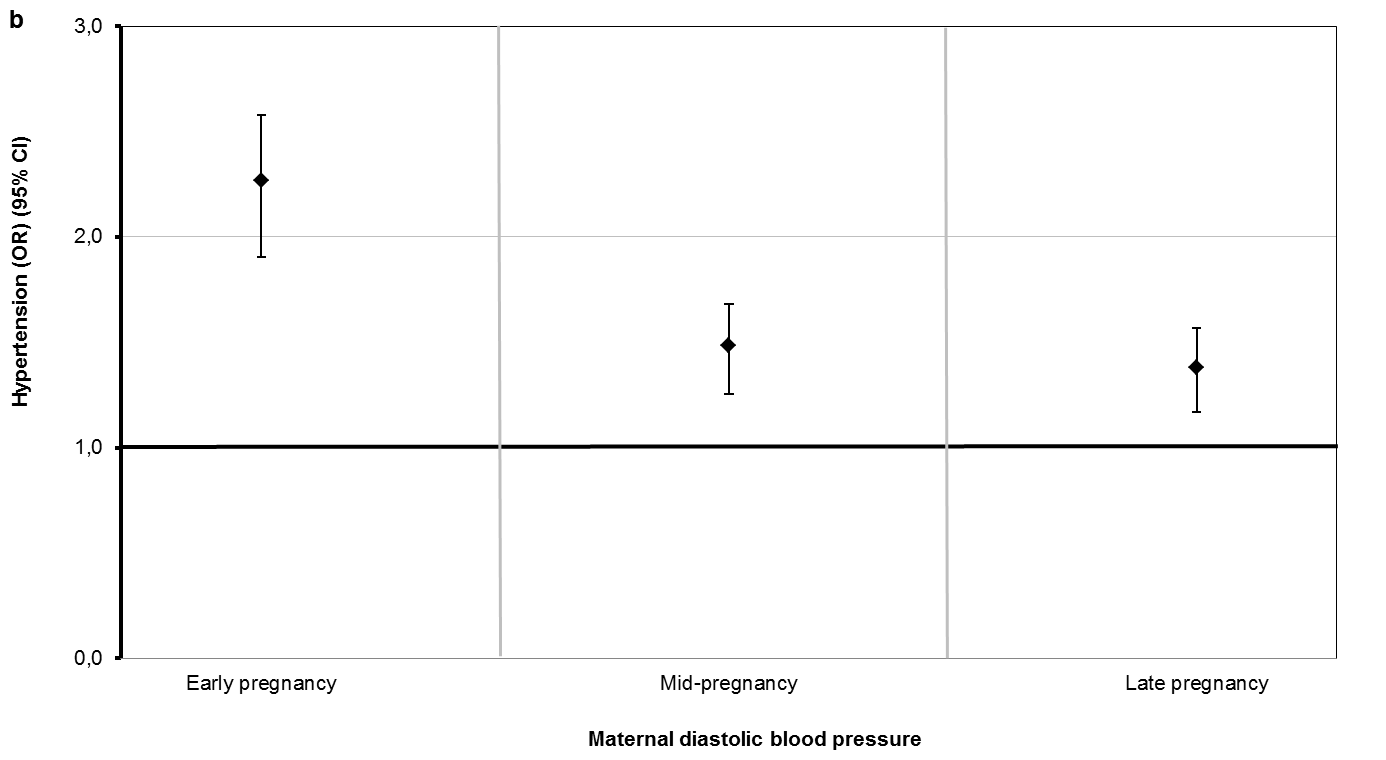


*Values are regression coefficients (95% Confidence Interval) from multivariable logistic regression models and reflect the difference in the risk of hypertension at follow-up per SDS change in early pregnancy systolic and diastolic blood pressure and per SDS change in standardised residual change in systolic and diastolic blood pressure in mid- and late pregnancy from conditional regression models (see for details of conditional regression models* ***Supplementary Information S3****). Models are adjusted for maternal age, visit interval, ethnicity, educational level, smoking, subsequent pregnancies between index and follow-up and child’s sex.*
