# Supplementary material for: Hypertensive disorders of pregnancy and subsequent maternal cardiovascular health
Source: Eur J Epidemiol. 2018 May 19;33(8):763–71. doi: 10.1007/s10654-018-0400-1 (PMC6061134; doi:10.1007/s10654-018-0400-1)
Supplement: Supplementary file 6 — Supplementary material 6 (DOCX 31 kb) [file 10654_2018_400_MOESM6_ESM.docx]

**Supplementary Information S6** Associations of hypertensive pregnancy disorders with the risk of hypertension six years after pregnancy (n = 4912)

| **Outcome** | **Normotensive pregnancies** | **GH** | | **PE** | |
| --- | --- | --- | --- | --- | --- |
|  | n =4612 | n = 205 | | n = 95 | |
|  |  | Odds ratio (95% CI)^†^ | Odds ratio (95% CI)^††^ | Odds ratio (95% CI)^†^ | Odds ratio (95% CI)^††^ |
| **Hypertension^†^** |  |  |  |  |  |
| Basic model | Reference | 5.8 (4.1-8.3)^**^ | 5.4 (2.9-10.0)^**^ | 4.4 (2.6-7.6)^**^ | 5.6 (2.4-13.5)^**^ |
| Confounder model | Reference | 6.6 (4.6-9.5)^**^ | 7.2 (3.7-13.8)^**^ | 4.5 (2.6-7.8)^**^ | 5.8 (2.4-14.3)^**^ |
| BMI model | Reference | 4.7 (3.2-6.9)^**^ | 5.5 (2.8-11.0)^**^ | 3.5 (1.9-6.2)^**^ | 4.9 (2.0-12.3)^**^ |

*Values are odds ratios and are based on logistic regression models. Estimates are from multiple imputed data.*

***Basic model****: Adjusted for maternal age at intake and visit interval;* ***Confounder model****: basic model and additionally adjusted for ethnicity, educational level, smoking, subsequent pregnancies between index and follow-up, and child's sex;* ***BMI model****: confounder model and additionally adjusted for BMI at follow-up. ^†^Defined as women using anti-hypertensive medication and/or having, in two subsequent readings, a systolic or diastolic blood pressure above 140 mmHg or 90mmHg, respectively. ^††^Defined as women using anti-hypertensive medication. ^*^P < 0.05, ^**^P < 0.01*
